# The Carbohydrate Threshold in Pregnancy and Gestational Diabetes: How Low Can We Go?

**DOI:** 10.3390/nu13082599

**Published:** 2021-07-28

**Authors:** Arianne Sweeting, Jovana Mijatovic, Grant D. Brinkworth, Tania P. Markovic, Glynis P. Ross, Jennie Brand-Miller, Teri L. Hernandez

**Affiliations:** 1 Sydney Medical School, The University of Sydney, Sydney, NSW 2006, Australia; arianne.sweeting@sydney.edu.au (A.S.); tania.markovic@sydney.edu.au (T.P.M.); glynis.ross@health.nsw.gov.au (G.P.R.); 2 Charles Perkins Centre, Boden Initiative, University of Sydney, NSW 2006, Australia; jovana.mijatovic@ctc.usyd.edu.au; 3 Department of Endocrinology, Royal Prince Alfred Hospital, Sydney, NSW 2050, Australia; 4 NHMRC Clinical Trials Unit, University of Sydney, Sydney, NSW 2006, Australia; 5 Commonwealth Scientific and Industrial Research Organisation—Health and Biosecurity, Sydney, NSW 2113, Australia; Grant.Brinkworth@csiro.au; 6 School of Health Sciences, The University of Sydney, Sydney, NSW 2006, Australia; 7 Metabolism & Obesity Services, Royal Prince Alfred Hospital, Sydney, NSW 2050, Australia; 8 School of Life and Environmental Sciences and Charles Perkins Centre, The University of Sydney, Sydney, NSW 2006, Australia; jennie.brandmiller@sydney.edu.au; 9 College of Nursing, Anschutz Medical Campus, University of Colorado, Aurora, CO 80045, USA; 10 Department of Medicine, Division of Endocrinology, Metabolism, and Diabetes, Anschutz Medical Campus, University of Colorado, Aurora, CO 80045, USA; 11 Children’s Hospital Colorado, Aurora, CO 80045, USA

**Keywords:** pregnancy, low carbohydrate, birth weight, micronutrients, ketones, lipids

## Abstract

The original nutrition approach for the treatment of gestational diabetes mellitus (GDM) was to reduce total carbohydrate intake to 33–40% of total energy (EI) to decrease fetal overgrowth. Conversely, accumulating evidence suggests that higher carbohydrate intakes (60–70% EI, higher quality carbohydrates with low glycemic index/low added sugars) can control maternal glycemia. The Institute of Medicine (IOM) recommends ≥175 g/d of carbohydrate intake during pregnancy; however, many women are consuming lower carbohydrate (LC) diets (<175 g/d of carbohydrate or <40% of EI) within pregnancy and the periconceptual period aiming to improve glycemic control and pregnancy outcomes. This report systematically evaluates recent data (2018–2020) to identify the LC threshold in pregnancy in relation to safety considerations. Evidence from 11 reports suggests an optimal carbohydrate range of 47–70% EI supports normal fetal growth; higher than the conventionally recognized LC threshold. However, inadequate total maternal EI, which independently slows fetal growth was a frequent confounder across studies. Effects of a carbohydrate intake <175 g/d on maternal ketonemia and plasma triglyceride/free fatty acid concentrations remain unclear. A recent randomized controlled trial (RCT) suggests a higher risk for micronutrient deficiency with carbohydrate intake ≤165 g/d in GDM. Well-controlled prospective RCTs comparing LC (<165 g/d) and higher carbohydrate energy-balanced diets in pregnant women are clearly overdue.

## 1. Introduction

Nutrition therapy remains the foundation of GDM treatment. A recent meta-analysis [1] showed that enhancing nutritional quality after GDM diagnosis, irrespective of the specific dietary approach, improved maternal fasting and postprandial glycemia, and reduced excessive birthweight (BW). However, to date no nutritional strategy in GDM has completely normalized maternal and fetal outcomes [2,3]. The original nutritional approach, which lowered total carbohydrate intake to 33–40% of total energy (EI) [4], reduced postprandial glycemia and fetal overgrowth patterns (defined as macrosomia [BW > 4000 g], large-for-gestational-age [LGA] or increased adiposity). Conversely, a separate body of evidence suggests higher carbohydrate intakes (60–70% EI) incorporating high quality carbohydrate sources of lower glycemic index and added sugars can also control maternal glycemia [5,6,7,8]. This suggests a range of nutritional patterns can be effective in GDM management, enabling personalized interventions [2] that optimize adherence and reduce the need for adjunctive medication.

Currently, the IOM recommends at least 175 g/d of carbohydrate intake during pregnancy [9]. However, many women are consuming lower carbohydrate (LC) diets during pregnancy and the periconceptual period [10,11,12,13] (<175 g/d [9] or <40% of EI [14]) in an attempt to improve glycemic control and pregnancy outcomes, but it remains unclear if this dietary practice is safe and appropriate to support maternal metabolic needs and optimal fetal growth. Safety concerns of LC diets in pregnancy relate to four key factors: (1) maintenance of the maternal/fetal glucose concentration gradient, relating to fetal growth and brain development [9,15,16]; (2) increased fetal exposure to maternal ketones, linked to impaired fetal and postnatal neural development [17,18,19,20,21]; (3) micronutrient deficiency risk [22,23]; and (4) risk of fetal exposure to increased maternal triglycerides (TG) or free fatty acids (FFA), linked to overgrowth [24,25,26,27,28]. In our view, there is a critical need to establish an acceptable LC intake threshold in pregnancy and GDM that safely supports maternal and fetal metabolic needs. This report systematically evaluates recent available data (2018–2020) to identify a LC threshold in pregnancy in relation to safety considerations.

## 2. Materials and Methods 

To accelerate translation of newly generated knowledge into clinical practice, this report evaluates findings from the most recently published clinical trials and cohort studies [29]. Four specific questions based on safety concerns related to LC diets in pregnancy (listed below), guided our literature search. Reports published between 2015–2020 were initially identified in PubMed using keyword combinations including: “low carbohydrate”, “pregnancy”, “fetal growth”, “high fat diet”, “fetal growth”, “diet pattern”, “birth weight (BW)”, “fetus”, “maternal ketones”, “betahydroxybutyrate”, “gestational diabetes”, “carbohydrate intake”, “micronutrients”. To be included, reports were then limited to those published between 2018–2020 [29]. Reports were published in English, were human studies in GDM or normal pregnancy, and represented original randomized controlled trials (RCTs) or oTablebservational investigations. Review papers and systematic reviews were excluded. A reported validated measure of dietary carbohydrate intake was necessary to evaluate if intake was related to fetal growth, ketone exposure, micronutrient deficiency, or increased maternal TG or FFA.

**Figure 1:**
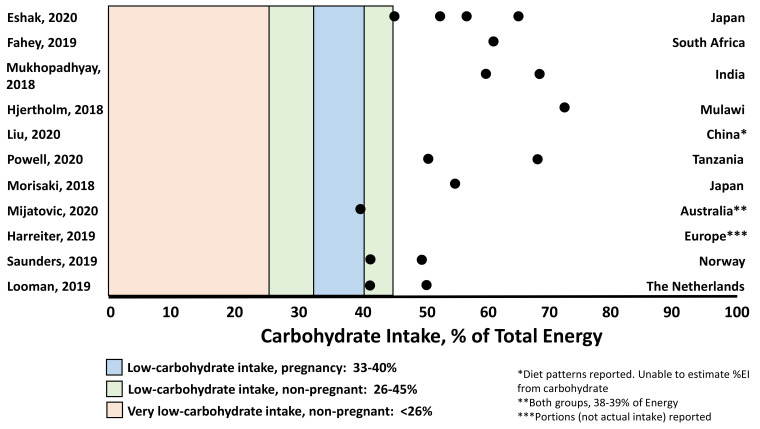
Energy intake from carbohydrate represented across 11 included studies in the context of definitions of low-carbohydrate intake within [4] and outside [41] of pregnancy (American Diabetes Association consensus statement). Unless reported in Table 1, Table 2, Table 3 and Table 4, energy from carbohydrate was calculated based on 4 kcal/g as a percentage of total EI. (1 kcal = 4.184 kJ).

A total of 1398 abstracts and/or full text manuscripts were screened. After elimination of duplicates, and studies that did not meet inclusion criteria, 11 reports met inclusion criteria (Table 1, Table 2, Table 3 and Table 4). Of these, 9 reported data from observational cohort or cross-sectional studies [30,31,32,33,34,35,36,37,38], 1 from an original RCT [39], and 1 from a secondary analysis of an RCT [40]. Figure 1 shows the distribution of carbohydrate intakes reported across these studies. Results are presented in alignment with the safety questions that guided the literature search. Historical context for each safety consideration is provided as further background.

## 3. Results & Discussion

### 3.1. Question 1: Does a LC Diet in Pregnancy Compromise the Maternal-Fetal Glucose Concentration Gradient?

Background. The human fetus relies on glucose for ~80% of its energy requirements with maternal glucose as the dominant substrate that supports growth and brain development [15]. Glucose moves across the placenta via facilitated diffusion, dependent on a higher maternal concentration relative to the fetus [42]. The IOM guideline for carbohydrate intake of ≥175 g/d is based on the estimated average requirement (EAR; to meet the needs of ~50% of a population) for carbohydrate outside of pregnancy (100 g/d), with an additional 33 g to support fetal brain development. When calculated as the recommended daily allowance (RDA; to meet the needs of 97–98% of a population), the following equation establishes the carbohydrate intake recommendation in pregnancy: (100 g/d [EAR outside of pregnancy] + 33 g [fetal brain development; rounded to 35 g]) + 2× population coefficient of variation at 15% = 175 g/d [9]. Recent evidence suggests the human placenta consumes more glucose than originally assumed [16], a factor not considered within the IOM recommendation. Theoretically, if the maternal diet is too low in carbohydrate and maternal glucose levels remain low, the plasma maternal-fetal glucose gradient may be compromised, jeopardizing fetal growth. Models of maternal undernutrition have demonstrated that inadequate total maternal EI is tightly associated with reduced and stunted fetal growth patterns [43], but it is often not possible to separate the role of insufficient energy vs. carbohydrate intake. Moreover, both restricted and very high protein intake has been associated with low birth weight [44,45,46].

Review. Several studies suggest that birth size varies by carbohydrate intake. In a Japanese cohort of 78,793 (Table 1) [30], women were categorized into quartiles of carbohydrate intake (45.1% to 64.9% of EI) and total EI. BW was lowest in the lowest quartile for both dietary factors. Birth length increased and ponderal index decreased with increasing carbohydrate intake. Similarly, the highest incidence of low BW (<2500 g) was observed in the lowest EI quartile based on a prior analysis of an expanded cohort (*n* = 91,637) [36] that showed neonates of women who consumed <47% of EI from carbohydrate had the lowest BWs, independent of total EI (Table 1). Importantly, higher fat intake >35% of EI, which often parallels LC intake, was also associated with lower BW. In South Africa, where carbohydrate intake follows a cyclical pattern with rainfall, harvest, gardening, and lean seasons [31], birth size z-scores increased with higher levels of carbohydrate consumption. However, whether total EI also fluctuated seasonally was unclear. Among pastoralist pregnant women in Tanzania [35], reduced EI during late pregnancy is a cultural tradition observed to prevent delivery complications related to larger BW. Women were found to reduce their EI by nearly 50% and carbohydrate intake by 64% to ~100 g/d. Compared to infants born in urban Tanzania where EI remained constant, BWs < 2500 g were higher (31% vs. 12%). Interestingly, brain growth assessed by head circumference (HC) in relation to total weight was compromised. BWs were lower but HCs were even lower (1.7 SD) than the WHO standard, and 40% (*n* = 46) were microcephalic (vs. *n* = 2 infants in urban comparison group). Similarly, an RCT comparing a LC intake (165 g/d) vs. routine care (190 g/d) in GDM (Table 1) [39], showed no differences in BW, body composition, small-for-gestational-age (SGA) or LGA incidence. However, neonates of women in the LC group had smaller HCs (adjusted for weight gain, gestational age, and infant sex), that could also have been related to lower total EI in the LC group. In contrast, a multi-site RCT secondary analysis [40] of a Healthy Eating (HE) lifestyle modification starting at <20 weeks’ gestation in women with obesity showed a reduced carbohydrate portion intake (g/d, %EI not reported) did not result in differences in BW, LGA, or SGA. Overall, only the data from Japan [36] suggest a LC threshold of <45–47% independent of total EI was associated with reduced fetal growth. Importantly, across studies (Figure 1) LC intakes tended to be accompanied by lower total EIs, a confounding factor that must be controlled to evaluate the independent effects of LC intake on fetal growth. No study reported maternal glucose concentrations.

Recently published data reveal other patterns of carbohydrate intake related to fetal growth. A cohort study in China (*n* = 7194) [34] showed a diet pattern lowest in carbohydrate (%EI not reported), with higher protein and fat, explained 40% of the variance in BW after adjustment for total EI and multiple confounders. BW was higher and SGA risk was lowest in those most adherent to the LC diet pattern (Table 1). Conversely, in India (*n* = 1837) [32], women who consumed >70% of energy from carbohydrate had the highest SGA rate (29%; male infants) after controlling for EI. In Malawi, carbohydrate intake >72% was negatively associated with HC (adjusted for total EI) (Figure 1) [33]. Importantly, in the cohorts from India and Malawi, higher carbohydrate intake was accompanied by modestly lower protein intake (11.5% and ~10%, respectively) [32,33]. This could be a potential factor in the SGA observations [44], although statistically protein intake was not associated with fetal growth. Collectively, the data suggest that a LC diet pattern may support appropriate fetal growth, but further studies are needed to separate the effects of LC from low EI. Moreover, carbohydrate intake as high as 70% can be a surrogate for poor diet quality, rich in foods with high glycemic index carbohydrates and added sugars [47], and the effect of diet quality remains unclear. Nonetheless, if low EI and LC intake are interlinked (as weight loss studies suggest) [41,48], caution is needed in pregnancy.

### 3.2. Question 2: In Pregnant Women Who Consume a LC Diet, Is There Greater Fetal Exposure to Maternal Ketones?

Background. Maternal ketones often increase in normal pregnancy, leading to high fetal ketone levels via passive diffusion across the placenta [49]. Maternal ketogenesis is most evident in later pregnancy due to increased lipolysis and fetal energy demand [19]. Carbohydrate restriction may also promote maternal ketonemia by increasing the ratio of glucagon to insulin, promoting oxidation of FFA to betahydroxybutyrate and other ketones. A safety concern for a LC diet in pregnancy is the potential risk of higher fetal exposure to maternal ketones. Early epidemiological studies evaluated the impact of fetal exposure to maternal ketones secondary to energy restriction, with no consistent association between ketonemia or ketonuria and poor fetal outcomes [50]. However, a seminal prospective US cohort study (*n* = 223, pre-existing diabetes, GDM or normal glucose tolerance) demonstrated an inverse correlation between higher maternal third trimester betahydroxybutyrate and FFA and lower offspring intellectual development scores at 2–5 years [17]. Measures of glycemic control did not correlate with cognitive scores, but total carbohydrate and EI, and maternal body mass index (BMI kg/m^2^) were not reported. Energy restriction (1200 kcal/d [50% carbohydrate, 30% fat, 20% protein]) in 12 women with GDM and obesity over the course of 1-week increased betahydroxybutyrate and ketonuria (vs. 2400 kcal/d diet) [20]. Ketonuria was also observed in some women consuming the control diet. In a separate study, reducing energy intake by 50% caused a 2.7-fold increase in betahydroxybutyrate and 2-fold increase in ketonuria (1200 kcal/d) with no detectable changes with 33% energy restriction (1600–1800 kcal/d) [21]; however, fetal outcomes were not reported. Despite the limitations of the historical data, fetal exposure to maternal ketones remains a safety concern in pregnancy.

*Review.* In a secondary analysis of a multi-site HE RCT [40] (Table 2), lower carbohydrate portions were associated with higher betahydroxybutyrate (0.082 vs. 0.068 mmol/L; *p* < 0.05) and higher fasting glucose (4.7 vs. 4.6 mmol/L; *p* < 0.05) at 24–28 weeks’ gestation (after ~4–8 weeks of HE), but not at 35–37 weeks’ gestation. As expected, carbohydrate intake at 24–28 weeks’ gestation was negatively correlated with betahydroxybutyrate. However, an RCT of LC intake (165 g/d) vs. routine care (190 g/d) (Table 2) in 46 women with GDM reported no increase in maternal betahydroxybutyrate levels over 6 weeks [39]. However, only 20% of women in the LC group achieved the prescribed 135 g/d target despite a lower EI (7040 vs. 8230 kJ). Thus, the effects of a LC intake <165 g/d in GDM on maternal ketonemia and fetal outcomes remain unclear.

### 3.3. Question 3: Do Pregnant Women Who Consume a LC Diet Have an Increased Risk for Micronutrient Deficiency?

Background. Recent cohort studies suggest maternal micronutrient intakes are commonly below recommended guidelines [37,38,39]. Maternal micronutrient deficiency may contribute to adverse fetal development and chronic disease via direct effects on hormonal adaptation and epigenetic gene regulation [51]. The potential for a LC diet to magnify micronutrient deficiency is a commonly cited concern. In the pre-conception period, a restricted carbohydrate intake (defined as ≤5th percentile among a control population, ~95 g/d) has been associated with neural tube defects [10], potentially independent of folic acid deficiency [11].

Review. An RCT conducted in women with GDM showed compared to a routine care diet containing 190 g/d of carbohydrate, a LC diet (165 g/d) achieved lower iron and iodine intakes from food (iron: 8.7 ± 0.4 vs. 10.6 ± 0.4 mg/d, *p* < 0.01; iodine: 147 ± 11 vs. 196 ± 14 μg/d, *p* < 0.01) (Table 3, Figure 1) [39]. Both iron and iodine decreased from pre- to post-study intervention in the LC group, suggesting a LC intake may promote an increased risk of micronutrient deficiency, particularly without supplement use. In a large Danish study [37], 44% of pregnant women reported carbohydrate intake below the recommended intake (RI) of 45–60% EI. Inadequate micronutrient intakes of folate (54% of women), iron (50%), calcium (36%), vitamin D (29%), iodine (24%) and selenium (41%) were also reported. However, whether the women with carbohydrate intake below the RI had higher incidence of micronutrient deficiencies was unknown. A separate study in Dutch women (*n* = 105) consuming moderate amounts of carbohydrate (range 43.2–49.7%) [38], showed that iron intake was inversely associated with glucose status. Folate, vitamins B6 and D intakes significantly changed through pregnancy, but were explained by supplement use rather than carbohydrate intake. In Japan (*n* = 78,793), median carbohydrate intake was 55% of EI, with most women consuming less than the Japanese recommendation (57.5% EI) [30]. Micronutrient intakes below recommendations were also reported (Table 3), suggesting micronutrient deficiencies may exist even with higher carbohydrate intake. Ponderal index was reduced across increasing quartiles of most micronutrients. Taken together, studies suggest micronutrient deficiency is common in pregnant women, and may be independent of dietary carbohydrate intake.

### 3.4. Question 4: Do Pregnant Women Who Consume a LC Diet Have Higher TG or FFA, Increasing Fetal Exposure to Lipids?

Background. While glucose is a dominant substrate for fetal growth, mounting evidence suggests a positive association between maternal lipids (TG, FFA) and fetal overgrowth [53]. Indeed, some reports demonstrate a more robust association between maternal TG and fetal growth than maternal glucose [54]. At the Fifth International Workshop on GDM in 2005, nutrition therapy recommendations focused only on carbohydrate restriction were revoked [25]. However, the role of maternal lipids in fetal growth remains unclear [55] despite the association of maternal FFA and fetal overgrowth [24,53]. In addition to a reduction in carbohydrate content, low carbohydrate diets also typically increase and have relatively higher proportions of daily caloric intake from fats (55–65%) and/or protein (25–30%) [56]. Specific to pregnancy, there is concern that higher dietary fat intake could increase maternal TG and FFA, potentially increasing fetal exposure to maternal lipids secondary to heightened maternal insulin resistance, leading to overgrowth [57]. Fetal exposure to increased maternal lipids, particularly through high fat diets, has been linked with developmental programming of offspring obesity and fatty liver in animal models and in human cohorts [44,57,58,59,60,61].

Review. In a secondary analysis of a multi-site RCT (Table 4) [40], women in the lower carbohydrate portion group (vs. higher carbohydrate) had higher fasting FFA and glucose after 4–8 weeks of intervention. At 35–37 weeks’ gestation, only FFA remained higher. At 24–28 weeks’ gestation, weak negative associations between carbohydrate intake and fasting FFA (r = −0.12, *p* < 0.03) and fasting glucose (r = −0.11, *p* < 0.03) were reported. There were no differences in cord blood C-peptide or fetal growth, and maternal lipids were not correlated with fetal growth. Future investigations are needed to establish the effects of higher fat intake on maternal TG/FFA concentrations and fetal growth.

## 4. Conclusions

Guided by four questions based on safety considerations related to LC diets during pregnancy, we set out to systematically evaluate recently published evidence to identify an acceptable LC intake threshold in pregnancy (Figure 1), if apparent. Available evidence suggests an optimal carbohydrate range between 47–70% EI supports normal fetal growth. Importantly, both the lower carbohydrate threshold of 47% EI, supported by data from a study of nearly 100,000 women [36], and the upper carbohydrate threshold of 70% EI [32,33], were independent of total EI. The lower threshold is higher than the conventionally recognized LC threshold of 33–40% EI in pregnancy [4]. Across studies, fetal growth tracked consistently with carbohydrate intake such that BWs are lower and incidences of SGA are higher with lower carbohydrate intake. While several studies did not control for total EI [30,31,35], the same response was observed in the large cohort study from Japan which controlled for EI [36]. The effects of a carbohydrate intake below the IOM recommendation of 175 g/d remain unclear, particularly intakes below the threshold at which women may experience ketonemia (<50 g/d outside of pregnancy) [39,62,63]. A major confounder in studies is the concurrent reduction in total EI with LC intake, a factor that independently impairs fetal growth [43] and promotes maternal ketonemia [20]. Caution should therefore be applied because inadvertent adverse effects of LC with or without caloric restriction could be severe. For example, an in vitro study of trophoblasts cultured from first trimester chorionic villi demonstrated that ketones suppress trophoblast uptake of glucose, jeopardizing glucose transfer across the placenta [18]. Evidence evaluated here showed reductions in head circumference occurred following ~50% maternal energy restriction with 100 g carbohydrate/d [35].

The reported high prevalence of poor maternal micronutrient intake may be independent of carbohydrate intake, although LC diets (<165 g/d) may exacerbate existing deficiencies [39]. Supplementation remains a key determinant of achieving sufficient micronutrient levels but may not always be accessible. The impact of a LC high-fat diet on maternal TG independent of the TG-raising effects of placental estrogen remains unclear [57]. Moreover, gestational weight gain is a strong independent predictor of fetal growth, requiring consideration along with carbohydrate and EI.

This systematic review and analysis of contemporary data had several strengths and limitations. Women enrolled across the 11 studies provided wide geographic representation of ethnicities in both developed and less developed countries. Further, sample sizes in the cohort studies were large and the analyses robust, with adequate control for confounding variables in most cases. Unfortunately, maternal glucose and ketone concentrations were not reported in relation to carbohydrate intake in the large cohort studies, and indicators of diet quality were lacking. It was also not possible to consider the effect of maternal obesity; in fact, many of the women were of normal weight across studies (Table 1, Table 2, Table 3 and Table 4). Although several studies included women who consumed ~40% of EI from carbohydrate [37,38,39], studies with the largest samples included women in higher carbohydrate intake ranges. No studies included women who consumed a very low carbohydrate diet (Figure 1). Although in most of the studies protein intake was not related to fetal growth, higher and lower intakes are known to be related with growth restriction [44]; future studies are required to evaluate the effects of a LC diet with higher protein intake. Optimal protein and fat intake in pregnancy were not the focus of this review per se. Explicit evaluation of the independent effects of glucose load and GI on maternal and fetal outcomes is also an important area for future investigation. Finally, few studies included women with GDM, in whom nutrition therapy is first-line treatment.

In conclusion, these data suggest that a carbohydrate intake in pregnancy between 47–70% supports normal fetal growth patterns. Due to the growing number of women with and without diabetes following a LC diet before and during pregnancy, well-controlled prospective RCTs and dose response studies examining the effects of energy-balanced dietary patterns with varying carbohydrate levels and specifically LC diets are clearly overdue.

## Figures and Tables

**Table 1:** Reports relevant to research question 1: Does a LC diet in pregnancy compromise the maternal-fetal glucose concentration gradient, suggested by reduced fetal growth and/or size at birth?

Report	Study Design Population	Carbohydrate Intake: Measurement and Amount	Statistical Adjustment	Carbohydrate Relationship to Perinatal Concern
Eshak, 2020 [30]	Observational birth cohort *n* = 78,793 Healthy pregnant women (39.6% primiparous, 31 ± 5 years) 78% had BMI 18.5 to <25 kg/m^2^ Mean gestational age at delivery 38.9 ± 1.5 weeks -Mean birth weight reported in right column Japan (15 regions represented)	Food frequency questionnaire Trimester 2 Median (IQR) CHO intake 223.8 (182.6–272.4) g/d 55.3% of total energy intake 61.9% of women consumed <recommended amount of CHO/d (57.5%) Quartiles of CHO% Intake Q1: 45.1% (1075 kcal/d) Q2: 52.9% (1466 kcal/d) Q3: 57.7% (1800 kcal/d) Q4: 64.9% (2650 kcal/d)	Geometric means of nutrients adjusted for: Maternal age Height Education Household income Pre-pregnancy BMI Net weight change in pregnancy Smoking Alcohol Thyroid disease Use of folate supplement Offspring sex, gestational age at delivery	CHO and total energy intake were associated with fetal growth (fully adjusted models) Q1-3 CHO%: Increased birth weight by quartile 3030 g → 3031 g → 3037 g → 3030 g (*p* = 0.07) Q1-Q4 CHO%: Increased birth length and decreased ponderal index by quartile (*p* = 0.002, *p* = 0.02, respectively) Q1-Q4 kcal/d: Increased birth weight by quartile 3026 g → 3031 g → 3036 g → 3036 g (*p* = 0.004) * 83.9% of women consumed <2500 kcal/d (recommended amount) Birth weight < 2500 g by energy intake quartile 8.4% → 7.6% → 7.2% → 7.1% (*p* < 0.001) -Fat intake was inversely associated with ponderal index (*p* = 0.05) -Protein intake was not associated with fetal growth
Fahey, 2019 [31]	Observational birth cohort *n* = 752 mother/infant dyads Pregnant women (43% primiparous, 14% HIV positive, 26.4 ± 6.3 years) Mean gestational age at delivery 39.3 ± 2.3 weeks Mean birth weight 3125 ± 452 g Vehmbe District, Limpopo Provence, South Africa	Food frequency questionnaire at delivery, to account for intake 1 month before delivery: 61 ± 10% CHO 24 ± 8.2% Fat 13 ± 2.9% Protein Rainfall: November–February Harvest: March–June Gardening: July–October Lean: November–February, ↑food insecurity	Models of dietary intake adjusted for: Maternal parity HIV status Height Education Marital status Household income Duration of pregnancy Models of birth size z-score adjusted for: Maternal parity HIV status Height Education Marital status Household income	CHO intake highest in lean season (64%, January) and lowest at end of Harvest (56%, June) Fat intake was lowest in lean (21%, January) and highest in Harvest season (28%, June) Birth size z-scores (weight, length, head circumference) peaked at lean season onset (November), declined, and were lowest at gardening season onset. Birth size scores tracked with seasonal CHO intake, where higher CHO intake was associated with higher birth size scores and vice versa
Mukhopadhyay, 2018 [32]	Observational birth cohort *n* = 1837 Healthy pregnant women (59% primiparous, 24.4 ± 3.8 years, BMI ~22 ± 4 kg/m^2^) Gestational age at delivery 38.6 ± 1.5 weeks Mean birth weight 2875 ± 450 g (28% SGA rate) -Women with an SGA infant were younger (0.5 year), shorter (0.1 m), weighed ~3 kg less and were more often primiparous) Bangalore, India	Food frequency questionnaire Trimester 1 Total energy: 1910 ± 517 kcal CHO: 64.6 ± 5.1% Fat: 23.9 ± 4.4% Protein: 11.5 ± 1.1% No differences in macronutrient intake between those with AGA vs. SGA infant Categories for low, adequate, high macronutrients: CHO: low < 60%, high > 70% Fat: low < 20%, high > 25% Protein: low < 10%, high > 20%	Macronutrient intakes adjusted for total energy intake (nutrient density method) AORs accounted for: Maternal age Education Parity Height Weight at recruitment Fetal sex Total energy intake	Male births only: Risk of SGA was higher with higher CHO intake (aOR per 5% energy: 1.15 [1.01–1.32]) Risk of SGA was lower with lower fat intake (aOR per 5% energy: 0.83 [0.71–0.97]) Categorical analysis In women with high CHO intake (≥334 g/d): 29% SGA rate aOR for SGA: 1.67 [1.002–2.780], *p* = 0.049 In women with high fat intake: 26% SGA rate aOR for SGA: 0.61 [0.41–0.90], *p* = 0.01 -This was only true for male infants
Hjertholm, 2018 [33]	Cross-sectional with random sampling *n* = 132 Maternal characteristics not reported Mean birth weight 3104 ± 401 g (6% had ‘low birth weight’) Nankumba Traditional Authority, Mangochi District, Malawi	Over a 10 d period: 3-d repeated interactive multi-pass 24-h recall Collected during post-harvest season, 28–35 weeks’ gestation (mean week of collection not reported) Median (IQR) intake: Energy: 2096.5 kcal (1778.1, 2570.6) CHO: 377 g (306, 454), ~72% Fat: 37.5 g (21.9, 51.7), ~16% Protein: 55 g (46, 67), ~10% ~1% of women consumed <135 g CHO/d and 60.6% consumed <59 g protein/d (both the estimated average requirement for pregnancy)	Associations adjusted for: Maternal age Weight Height Gestational age Literacy Marital status Household assets Parity Maternal energy intake Newborn gender	With each 1%↑ in fat intake, there was a 0.1 cm increase in birth length and abdominal circumference With each 1% ↑ in CHO intake, there was a 0.1 cm decrease in birth length and abdominal circumference CHO intake was negatively associated with head circumference (β ≤ −0.01, *p* = 0.04) [small effect] Adjusted for energy intake -Most CHO intake in this region is accounted for by nisma (porridge made from maize)
Liu, 2020 [34]	Cross-sectional *n* = 7194 Pregnant women (56.7% ages 25–34 years, 76.6% rural, 60.4% primiparous) Mean gestational age 39 ± 1 weeks Mean birth weight 3253.9 ± 448.3 g (z-score −0.07 [SD 1.15], SGA rate 13.2% Shaanxi Province, China	107-item semi-quantitative food frequency questionnaire -represented intake during all of gestation Response variables in relation to birth weight were: protein and CHO density (g/4184 kJ), ratio PUFA + MUFA: SFA, haem Fe density (mg/4184 kJ) 2 diet patterns (DP) identified explained 63.1% of variation in response variables): DP1: higher protein and haem Fe, lower CHO and higher fat density. ↑legumes, soyabean, vegetables, meat, dairy, eggs, fish; ↓wheat, oils (Explained 40.1% of total response variables; 13.1% of response variables explained by CHO) DP2: Lower protein, higher CHO, lower fat and haem Fe. ↑wheat, rice, potatoes, vegetables, fruit; ↓nuts, red meat, oils (Explained 23.0% of total response variables; 65.6% of response variables explained by CHO)	Model 1: Unadjusted Model 2: Total energy intake Maternal age Education Residence Per captia annual household income Model 3: Model 2 + Parity Smoking Passive smoking Alcohol Pregnancy consultation Number of antenatal visits Folic acid/Fe/ multiple-micronutrient supplementation Models for BW and LBW were also adjusted for sex, gestational age	Across low, medium, and high adherence to DP1: SGA incidence 15.1% → 13.0% → 11.7% (*p* = 0.002) Birth weight 3225 g → 3261 g → 3276 g (*p* < 0.001) Birth weight z-score −0.15 → −0.05 → −0.01 (*p* < 0.001) These associations were significant across fully adjusted models
Powell, 2020 [35]	Observational birth cohort *n* = 141 mother/infant dyads Maasai pastoralist women *n* = 102 neonates from Mwanza (urban/peri-urban center) Included for BW comparisons only. Women were not instructed to reduce EI, but intake not measured All births during the dry season (June–September) Gestational age at delivery not reported Ngorongoro Conservation Area (NCA), Northern Tanzania	Food frequency questionnaire developed/validated for this Maasai cohort Administered 2−3 d postpartum on 2 occasions to assess early-mid (T1-2) and 3rd trimester (T3) pregnancy, respectively Intake T1-2 Total Energy: 1601 ± 734.19 kcal/d CHO intake T1-2: 276.04 g/d [95% CI: 237.72−314.37] 76% of total Fat intake: 43.83 g/d (37.67−49.99) Protein: 45.27 g/d (8.69−51.86)	Adjustments: Traditional birth attendant	Intake change from T1-2 to onset of T3: Total energy: 1601 → 799 ± 317.59 kcal/d CHO: 276 → 100.27 g/d (95% CI: 62.46−138.08) Fat: 43.83 → 23.43 g/d (17.38−29.48) Protein: 45.27 → 30.17 g/d (23.69−36.65) Reductions were: Total energy: −902.35 ± 74.94 kcal CHO: −175.78 ± 13.14 g (64% of total) Fat: −20.397 ± 2.32 g Protein: −15.099 ± 2.47 g *p* < 0.01 for all Birth weight and head circumference z-scores in neonates from Mwanza and NCA fell below the WHO standard Head circumference in neonates from NCA were far lower (1.7 SD) than standard (<50%tile at 36 weeks’), more so than weight (>50%tile at 36 weeks’). 31% had birth weight <2500 g (vs. 12% Mwanza), 40% were microcephalic (vs. *n* = 2 Mwanza).
Morisaki, 2018 [36] (Same cohort as Eshak) [30]	Observational birth cohort *n* = 91,637 Healthy pregnant women (40.3% primiparous, 31 ± 5 years) 73.6% had BMI 18.5 to <25 kg/m^2^ Gestational age at delivery: >28 weeks and ≤42 weeks Mean birth weight 3028 ± 406 g (6.9% SGA) Japan (15 regions represented)	Food frequency questionnaire Early pregnancy (FFQ1) to represent previous year Mid-pregnancy to represent intake during pregnancy Intake at FFQ1 Total energy: 7475.1 ± 2575.7 kJ/d CHO: 243.4 ± 80.2 g/d (55.3%) Fat: 59.9 ± 28.4 g/d (29.5%) Protein: 61.2 ± 25.6 g/d (13.5%) Intake at FFQ2 Total energy: 7184 ± 2506 kJ/d CHO: 233.7 ± 77 g/d (55.3%) Fat: 58.2 ± 27.9 g/d (29.8%) Protein: 58.9 ± 25.1 g/d (13.6%)	For models where CHO or fat were used to predict fetal growth, adjusted for: total energy intake Protein intake CHO or fat intake (appropriate to model) Confounders: Maternal age Parity Education Income Pre-pregnancy BMI Height Smoking status Infant sex Adjustments for: recruitment site Total energy intake Gestational weight gain Age	FFQ1 and FFQ2 related to birth weight: Birth weight was highest with 12% protein even when isoenergetic replacement with CHO or fat was modeled. Lower birth weight with protein >14% U-shaped association between protein density and SGA risk. Lowest SGA risk with protein at 12% even when isoenergetic replacement with CHO or fat was modeled. Higher SGA risk if protein >15% Controlled for protein, energy intake and maternal characteristics: Fat (FFQ1) Fat density of 25% associated with highest birth weight. Fat density >35% associated with lower birth weight CHO (FFQ1) CHO density of 59% (~264 g/d) had highest birth weight. CHO density <47% (~210 g/d) had lower birth weight.
Mijatovic, 2020 [39]	Randomized Controlled Trial *n* = 46 Women with gestational diabetes diagnosed at ~20 weeks’ gestation (10–14% primiparous, 33.3 ± 0.6 year, BMI 26.8 ± 0.9 kg/m^2^) 28.5 ± 0.4 weeks’ gestation Modestly lower CHO: 135 g/d Routine Care: 180–200 g/d Mean gestational age at delivery: 38 ± 0.2 weeks Primary outcome: difference in blood ketones between diet groups Australia	24-h recalls 3 d food diaries Moderately lower CHO: 165 ± 7 g/d (20% achieved target) Energy intake: 7040 ± 240 kJ/d 25% insulin, 4% metformin Routine Care: 190 ± 9 g/d (65% achieved target) Energy intake: 8230 ± 320 kJ (*p* < 0.01) 31.8% insulin, 4.5% metformin Gestational weight gain similar (8–10 kg, *p* > 0.05)	Gestational weight gain Infant sex Gestational age at delivery Insulin status	No difference in birth weight, %fat, fat-free mass, LGA between groups Neonates in moderately lower CHO group had smaller head circumference (*p* = 0.04 after adjustment for weight gain, gestational age, infant sex) Intake differences from baseline → after 6 weeks: Moderately lower CHO: Energy: 7480 → 7040 kJ/d CHO: 167 → 165 g/d Fat: 74 → 71 g/d Protein: 100 → 85 g/d Routine care: Energy: 7510 → 8230 kJ/d CHO: 164 → 190 g/d (*p* = 0.04) Fat: 77 → 82 g/d (*p* > 0.05) Protein: 99 → 103 g/d (*p* < 0.01)
Harreiter, 2019 [40]	Randomized controlled trial Secondary analysis *n* = 436 Women with obesity <20 weeks’ gestation (~35% primiparous, ~32 ± 5 years, Pre-pregnancy BMI ~34 ± 4 kg/m^2^) Mean gestational age at delivery ~39 ± 2 weeks Mean birth weight ~3500 g Healthy eating: *n* = 221 No healthy eating: *n* = 215 ~20–22% GDM rate/group (*p* > 0.05) Nine European countries (86.7% of European descent)	12-item questionnaire, frequencies (days/wk) -Only portions recorded 24–28 weeks (HE—No HE, adjusted mean difference (95%CI)) Portion size: −2.8 (−5.4, −0.1) * CHO: −2.0 (−6.4, 2.3) Fat: −1.3 (−2.3, −0.2) Protein: 1.1 (−0.2, 2.4) 35–37 weeks Portion size: −3.8 (−6.8, −0.9) ** CHO: −6.2 (−11.6, −0.9) * Fat: −1.5 (−2.8, −0.3) * Protein: 0.3 (−1.2, 1.7) * *p* < 0.05 ** *p* < 0.01	Baseline level of outcome variable Or Baseline level of outcome variable + age + BMI at assessment date + gestational age + HOMA-IR + self-reported physical activity + self-reported food intake + smoking Gestational weight gain analyses adjusted for baseline BMI Dietary, physical activity analyses adjusted for baseline level	No differences in birth weight, LGA or SGA No difference in physical activity Weight gain (HE vs. No HE) 24–28 weeks’ gestation: 3.3 ± 2.7 vs. 4.3 ± 2.8 kg (*p* < 0.001) 35–37 weeks’ gestation: 7.0 ± 4.4 vs. 8.5 ± 4.7 kg (*p* < 0.01)

Eleven reports met criteria for inclusion shown in Table 1, Table 2, Table 3 and Table 4. Sample sizes ranged from *n* = 46 [39] to *n* = 91,637 [36] and represented pregnant women across a range of geographic regions, including Japan [30,36], China [34], South Africa [31], India [32], Malawi [33], Tanzania [35], Australia [39], Norway [37], the Netherlands [38], and across Europe [40].

**Table 2:** Reports relevant to research question 2: In pregnant women who consume a LC diet, is there greater fetal exposure to maternal ketones?

Report	Study Design Population	Carbohydrate Intake: Measurement and Amount	Statistical Adjustment	Carbohydrate Relationship to Perinatal Concern
Mijatovic, 2020 [39]	Randomized Controlled Trial *n* = 46 Women with gestational diabetes diagnosed at ~20 weeks’ gestation (10–14% primiparous, 33.3 ± 0.6 year, BMI 26.8 ± 0.9 kg/m^2^) 28.5 ± 0.4 weeks’ gestation Modestly lower CHO: 135 g/d Routine Care: 180–200 g/d Mean gestational age at delivery: 38 ± 0.2 weeks Primary outcome: difference in blood ketones between diet groups Australia	24-h recalls 3 d food diaries Moderately lower CHO: 165 ± 7 g/d (20% achieved target) Energy intake: 7040 ± 240 kJ/d 25% insulin, 4% metformin Routine Care: 190 ± 9 g/d (65% achieved target) Energy intake: 8230 ± 320 kJ (*p* < 0.01) 31.8% insulin, 4.5% metformin Gestational weight gain similar (8–10 kg, *p* > 0.05)	Gestational weight gain Infant sex Gestational age at delivery Insulin status	Moderately lower CHO vs. Routine Care (ketones < 0.5 mmol/L = normal) Average of fasting blood, pre-prandial lunch, dinner Baseline 0.1 ± 00 vs. 0.2 ± 00 mmol/L (*p* > 0.05) 6 weeks later 0.1 ± 0.0 vs. 0.1 ± 0.0 mmol/L (*p* > 0.05)
Harreiter, 2019 [40]	Randomized controlled trial Secondary analysis *n* = 436 Women with obesity <20 weeks’ gestation (~35% primiparous, ~32 ± 5 years, Pre-pregnancy BMI ~34 ± 4 kg/m^2^) Mean gestational age at delivery ~39 ± 2 weeks Mean birth weight ~3500 g Healthy eating (HE): *n* = 221 No healthy eating: *n* = 215 ~20–22% GDM rate/group (*p* > 0.05) Nine European countries (86.7% of European descent)	12-item questionnaire, frequencies (days/wk) -Only portions recorded 24–28 weeks (HE—No HE, adjusted mean difference (95%CI)) Portion size: −2.8 (−5.4, −0.1) * CHO: −2.0 (−6.4, 2.3) Fat: −1.3 (−2.3, −0.2) Protein: 1.1 (−0.2, 2.4) 35–37 weeks’ gestation Portion size: −3.8 (−6.8, −0.9) ** CHO: −6.2 (−11.6, −0.9) * Fat: −1.5 (−2.8, −0.3) * Protein: 0.3 (−1.2, 1.7) * *p* < 0.05 ** *p* < 0.01	Baseline level of outcome variable Or Baseline level of outcome variable + age + BMI at assessment date + gestational age + HOMA-IR + self-reported physical activity + self-reported food intake + smoking Gestational weight gain analyses adjusted for baseline BMI Dietary, physical activity analyses adjusted for baseline level	HE vs. No HE 24–28 weeks’ gestation Fasting blood beta-hydroxybutyrate: 0.082 ± 0.065 vs. 0.068 ± 0.067 (*p* < 0.05) 35–37 weeks’ gestation Fasting blood beta-hydroxybutyrate: 0.107 ± 0.071 vs. 0.101 ± 0.092

**Table 3:** Reports relevant to research question 3: Do pregnant women who consume a LC diet have an increased risk for micronutrient deficiency?

Report	Study Design Population	Carbohydrate Intake: Measurement and Amount	Statistical Adjustment	Carbohydrate Relationship to Perinatal Concern
Eshak, 2020 [30]	Observational birth cohort *n* = 78,793 Healthy pregnant women (39.6% primiparous, 31 ± 5 years) 78% had BMI 18.5 to < 25 kg/m^2^ Mean gestational age at delivery 38.9 ± 1.5 weeks -Mean birth weight reported in right column Japan (15 regions represented)	Food frequency questionnaire Trimester 2 Median (IQR) CHO intake 223.8 (182.6–272.4) g/d 55.3% of total energy intake 61.9% of women consumed <recommended amount of CHO/d (57.5%) Quartiles of CHO% Intake Q1: 45.1% (1075 kcal/d) Q2: 52.9% (1466 kcal/d) Q3: 57.7% (1800 kcal/d) Q4: 64.9% (2650 kcal/d) Proportion of women consumed < recommended amount of micronutrients Vitamin A 63% Vitamin K 48% Vitamin E 61% Vitamin D 87% Vitamin C 67%, Vitamin B6 73% Folate 88% Vitamin B12 26%	Geometric means of nutrients adjusted for: Maternal age Height Education Household income Pre-pregnancy BMI Net weight change in pregnancy Smoking Alcohol Thyroid disease Use of folate supplement Offspring sex, gestational age at delivery	Increasing quartiles of micronutrients: Vitamin C and folate intake associated with birthweight; Vitamins C, D, K, B6, B12 and folate associated with birth length Vitamins A, E and D associated with head circumference; Vitamins A, C and D associated with chest circumference Vitamin K inversely associated with the ponderal index in the offspring
Saunders, 2019 [37]	Observational birth cohort *n* = 1674 Healthy pregnant women (62.9% primiparous, 32.5 ± 4.1 years) BMI 24.6 ± 3.5 kg/m^2^ Recruited between 16–22 weeks’ gestation Norway	Food frequency questionnaire First half of pregnancy Total energy: 10,082 (4139) kJ CHO: 45.7 (42.3–49.2) % Fat: 34.5 (31.2–37.8) % Protein: 16.5 (15.1–18.1) % Below and above Recommended Intake Range for macronutrients: CHO: Below 43.9%, above 0.5% Fat: Below 2.9%, above 14.0% Protein: Below 0.2%, above 6.9% Micronutrients: Vit A: Below 9.6%, above 90.4% Vit C: Below 4.4%, above 95.6% Vit D: Below 28.7%, above 71.3% Vit B12: Below 0.3%, above 99.7% Iodine: Below 24.4%, above 75.6% Folate: Below 54.4, above 45.6% Zinc: Below 10.2, above 89.8% Ca: Below 36.2%, above 63.8% Selenium: Below 41.3%, above 58.7% Iron: Below 41.3%, above 58.7% Median (IQR) Based on Nordic Nutrition Recommendations, 2012 [52]	Educational level (post-hoc analysis)	No association between educational levels and micronutrient intake
Looman, 2019 [38]	Observational Birth Cohort *n* = 105 Preconception to <24 weeks’ gestation (32yo, 93% multiparous, median BMI preconception 24.4 kg/m^2^ The Netherlands	FFQ 75 g 2-h OGTT At pre-conception, 12 and 24 weeks’ gestation Energy intake increased during pregnancy from 8583 (6713; 9462) kJ at preconception to 9189 (7432; 10,541) kJ at 24 weeks’ gestation Median CHO 46.5% (43.2; 49.7) Preconception 45.4% (42.3; 48.6) TM1 46.5% (45.2; 50.3) TM2 48.1% (44.8; 50.3)	Covariates: Age Education Ethnicity Parity Smoking Nausea in pregnancy Vomiting in pregnancy Season of blood collection Physical Activity Energy intake Alcohol Time between measurements Hx of GDM BMI Adjusted for supplement intake	Iron intake inversely associated with fasting glucose and HbA1c Folate, vitamin B6 and vitamin D levels significantly changed through pregnancy, accounted for by intake of supplements
Mijatovic, 2020 [39]	Randomized Controlled Trial *n* = 46 Women with gestational diabetes diagnosed at ~20 weeks’ gestation (10–14% primiparous, 33.3 ± 0.6 years, BMI 26.8 ± 0.9 kg/m^2^) 28.5 ± 0.4 weeks’ gestation Modestly lower CHO: 135 g/d Routine Care: 180–200 g/d Mean gestational age at delivery: 38 ± 0.2 weeks’ Primary outcome: difference in blood ketones between diet groups Australia	24-h recalls 3d food diaries Moderately lower CHO: 165 ± 7 g/d (20% achieved target) Energy intake: 7040 ± 240 kJ/d 25% insulin, 4% metformin Routine Care: 190 ± 9 g/d (65% achieved target) Energy intake: 8230 ± 320 kJ (*p* < 0.01) 31.8% insulin, 4.5% metformin Gestational weight gain similar (8–10 kg, *p* > 0.05)	Gestational weight gain Infant sex Gestational age at delivery Insulin status	Moderately lower CHO: lower Fe, iodine

**Table 4:** Reports relevant to research question 4: Do pregnant women who consume a LC diet have higher TG or FFA, increasing fetal exposure to lipids?

Report	Study Design Population	Carbohydrate Intake: Measurement and Amount	Statistical Adjustment	Carbohydrate Relationship to Perinatal Concern
Harreiter, 2019 [40]	Randomized controlled trial Secondary analysis *n* = 436 Women with obesity <20 weeks’ gestation (~35% primiparous, ~32 ± 5 years, pre-pregnancy BMI ~34 ± 4 kg/m^2^) Mean gestational age at delivery ~39 ± 2 weeks Mean birth weight ~3500 g Healthy eating: *n* = 221 No healthy eating: *n* = 215 ~20–22% GDM rate/group (*p* > 0.05) Nine European countries (86.7% of European descent)	12-item questionnaire, frequencies (days/wk) -Only portions recorded 24–28 weeks’ gestation (HE—No HE, adjusted mean difference (95%CI)) Portion size: −2.8 (−5.4, −0.1) * CHO: −2.0 (−6.4, 2.3) Fat: −1.3 (−2.3, −0.2) Protein: 1.1 (−0.2, 2.4) 35–37 weeks’ gestation Portion size: −3.8 (−6.8, −0.9) ** CHO: −6.2 (−11.6, −0.9) * Fat: −1.5 (−2.8, −0.3) * Protein: 0.3 (−1.2, 1.7) * *p* < 0.05 ** *p* < 0.01	Baseline level of outcome variable Or Baseline level of outcome variable + age + BMI at assessment date + gestational age + HOMA-IR + self-reported physical activity + self-reported food intake + smoking Gestational weight gain analyses adjusted for baseline BMI Dietary, physical activity analyses adjusted for baseline level	HE vs. No HE 24–28 weeks’ gestation TG: 1.88 ± 0.63 vs. 1.85 ± 0.68 mmol/L FFA: 0.60 ± 0.19 vs. 0.55 ± 0.17 mmol/L (*p* < 0.01) Fasting glucose: 4.8 ± 0.4 vs. 4.6 ± 0.4 mmol/L (*p* < 0.05) 35–37 weeks’ gestation TG 2.42 ± 0.8 vs. 2.27 ± 0.8 mmol/L FFA: 0.64 ± 0.23 vs. 0.59 ± 0.21 (*p* < 0.05) Fasting glucose: 4.6 ± 0.5 vs. 4.5 ± 0.4 mmol/L
